# Mitochondrial genome of *Parasinilabeo longicorpus* provides insights into the phylogeny of Labeoninae

**DOI:** 10.1080/23802359.2020.1798298

**Published:** 2020-07-27

**Authors:** Xianyu Pan, Liyi Shao, Jianqiang Lao, Tianxu Kuang, Jiehu Chen, Lei Zhou

**Affiliations:** a Joint Laboratory of Guangdong Province and Hong Kong Region on Marine Bioresource Conservation and Exploitation, College of Marine Sciences, South China Agricultural University, Guangzhou, China; b Science Corporation of Gene, Guangzhou, China; c Guangdong Laboratory for Lingnan Modern Agriculture, Guangzhou, China

**Keywords:** *Parasinilabeo longicorpus*, mitochondrial genome, Labeoninae, karst region

## Abstract

*Parasinilabeo longicorpus* is an endemic species living in the karst regions of South China. In the present study, we firstly performed the complete mitogenome sequence of *P. longicorpus* (Cyprinidae: Labeoninae). Results showed that the mitogenome was 16,596 bp in length and contained 37 genes (i.e. 13 protein-coding, 22 transfer RNA, and two ribosomal RNA genes) and a single control region. The genetic arrangement and distribution of the mitogenome were consistent with that of other vertebrates. Phylogenetic analysis of the protein-coding genes indicated that Labeoninae species could be divided into three branches. *Parasinilabeo longicorpus* showed close evolutionary relationship with *P. assimilis*. This study should help to improve our understanding of the mitogenomes and phylogenetics of Labeoninae, and provide a foundation for further taxonomic, systematic, and genetic studies.

Karst, with high levels of endemic species, is one of the most threatened ecosystems in the world. Because of the unique geological structure, karst is very fragile and extremely sensitive to environmental changes (Zhao et al. 2011; Palandačić et al. 2012). However, the scarcity of available data on genetic information greatly limits the formulation of current biodiversity conservation strategies. Here, we sequenced, annotated, and characterized the complete mitochondrial genome (mitogenome) of *P. longicorpus*, an endemic fish distributed in the karst regions of southern China. We aimed to characterize the complete mitochondrial genome of the *P. longicorpus* and to explore its phylogenetic relationships within Labeoninae.

The specimen of *P. longicorpus* used in this study were collected with gill net in Lianzhou, Guangdong Province, China (24°46′53.9″N 112°22′25.1″E), in October 2018. It was maintained in South China Agricultural University with the accession no. SCAU-20181028001. The complete mitogenome was sequenced by next-generation sequencing using the Illumina HiSeq2500 instrument (Illumina, Inc., San Diego, CA, USA) with the de novo assembly strategy (Shao et al. 2020).

The complete mitogenome of *P. longicorpus* (GenBank accession number MN123555) is a closed-circular molecule of 16,596 bp in length and contains the typical set of 13 protein-coding genes (PCGs), two ribosomal RNA genes (*12S RNA* and *16S RNA*), 22 transfer RNA genes (tRNAs), and a single control region.

We established a phylogenetic tree based on the mitogenome protein-coding genes from 19 Labeoninae species and *Cyprinus carpio* as the outer group (Figure 1). The maximum-likelihood (ML) and Bayesian inference (BI) approaches demonstrated highly similar topologies and branch lengths. We found that the 19 Labeoninae species could be divided into three branches, with *Cyprinus carpio* as the outer group. *Parasinilabeo longicorpus* showed closest evolutionary relationship with *P. assimilis*. Clade A consisted of all species from China and contained most species and genera in Labeoninae. This clade could be further divided into four subgroups: Clade A1 contained *Ptychidio* and *Parasinilabeo* and Clade A2 included all species of *Discogobio*. Interestingly, all members of Clade A1 + Clade A2 were karst-distributed species from regions in southern China. Clade A1 + Clade A2 was the sister group of Clade A3, which included two species of Garra. Clade A4 included two species of *Bangana* and was the sister group to Clade A1 + Clade A2 + Clade A3.

**Figure 1.**
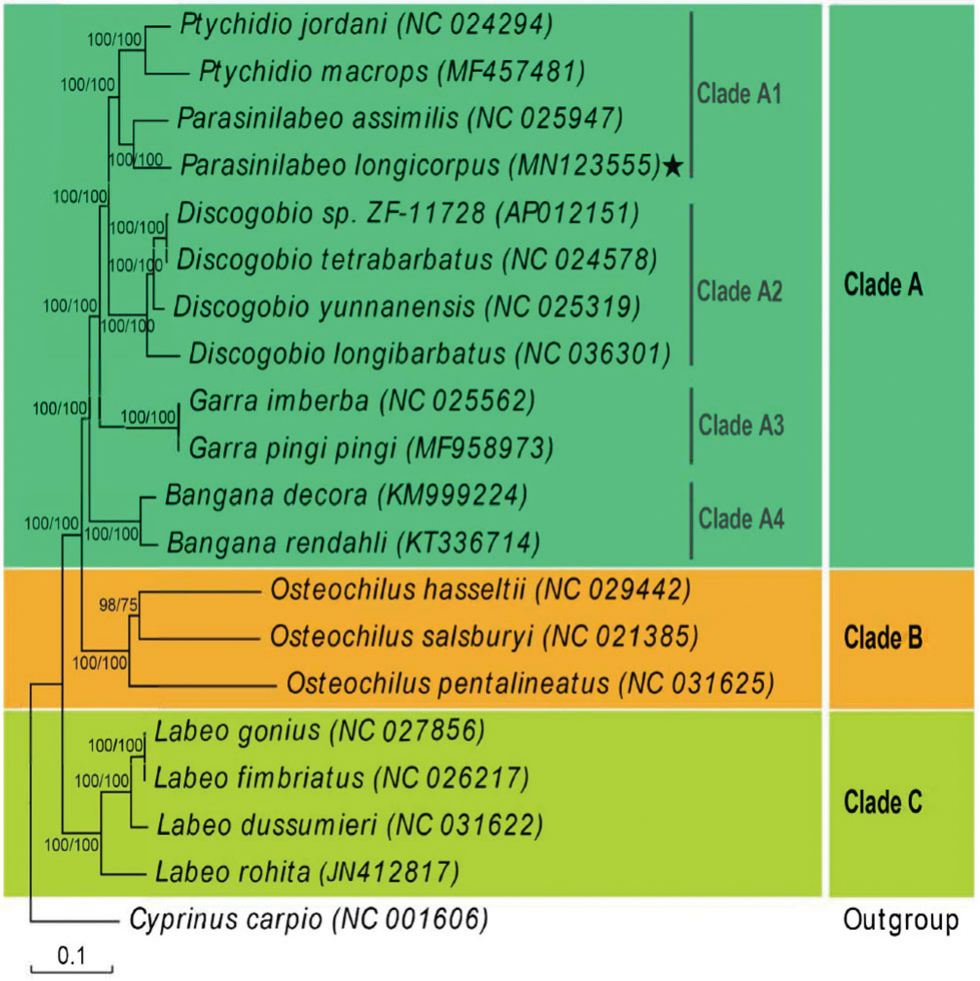
Phylogenetic tree of *Parasinilabeo longicorpus* based on sequences of 13 protein-coding genes. Numbers along nodes indicate posterior probabilities (BI) and bootstrap support (ML).

Our research results should help elucidate the mitogenomes and phylogeny of the Labeoninae subfamily and the species within it, which are essential aspects for future taxonomic, systematic, and genetic studies.

## Data Availability

The data that support the findings of this study are openly available in GenBank of NCBI at https://www.ncbi.nlm.nih.gov, reference number MN123555.
